# The Future Ethics of Artificial Intelligence in Medicine: Making Sense of Collaborative Models

**DOI:** 10.1007/s11948-022-00369-2

**Published:** 2022-04-01

**Authors:** Torbjørn Gundersen, Kristine Bærøe

**Affiliations:** 1 Centre for the Study of Professions, Oslo Metropolitan University, Oslo, Norway; 2 Department of Global Public Health and Primary Care, University of Bergen, Bergen, Norway

**Keywords:** Artificial intelligence, Machine learning, Medical ethics, Ethical design, Collaboration, Deliberation, Professional responsibility

## Abstract

This article examines the role of medical doctors, AI designers, and other stakeholders in making applied AI and machine learning ethically acceptable on the general premises of shared decision-making in medicine. Recent policy documents such as the EU strategy on trustworthy AI and the research literature have often suggested that AI could be made ethically acceptable by *increased collaboration* between developers and other stakeholders. The article articulates and examines four central alternative models of how AI can be designed and applied in patient care, which we call *the ordinary evidence model*, *the ethical design model*, *the collaborative model*, and *the public deliberation model*. We argue that the collaborative model is the most promising for covering most AI technology, while the public deliberation model is called for when the technology is recognized as *fundamentally transforming the conditions* for ethical shared decision-making.

## Introduction

Recent developments in artificial intelligence (AI) and machine learning, such as deep learning, has the potential to make medical decision-making more efficient and accurate. Deep learning technologies can improve how medical doctors gather and analyze patient data as a part of diagnostic procedures, prognoses and predictions, treatments, and prevention of disease (Becker, 2019; Ienca & Ignatiadis, 2020; Topol, 2019a, 2019b). However, applied artificial intelligence raises numerous ethical problems, such as the severe risk of error and bias (Ienca & Ignatiadis, 2020, p. 82; Marcus & Davis, 2019), lack of transparency (Müller, 2020), and disruption of accountability (De Laat, 2018). Describing the ethical challenges and concerns has so far been the main focus of the increasing research literature in general AI ethics (Müller, 2020) and ethics of medical AI (e.g., Char et al., 2018, 2020; Grote & Berens, 2019; McDougall, 2019; Vayena et al., 2018). Furthermore, if clinicians’ decisions are to be substantially assisted, or even replaced by AI and machine learning, *shared decision-making*—a central ethical ideal in medicine that protects patient autonomy by letting patients make informed choices about their healthcare in line with their values—is challenged. The opacity and dynamic nature of machine learning algorithms might undermine proper interaction between medical doctors and patients over the basis for a diagnosis and the choice of a treatment. This article examines the role of medical doctors, AI designers, and other stakeholders in making applied AI and machine learning ethically acceptable on the general premises of shared decision-making. Whether these premises should be reconfigured as AI develops is a separate ethical issue that we leave aside in this paper.

The severe ethical concerns over applied AI and machine learning have led to numerous ethical initiatives from governments, industry, NGOs, and academia seeking to formulate ethical principles that ensure ethically acceptable and trustworthy AI (for overviews, see Jobin et al., 2019; Schiff et al., 2020). Recent policy documents such as the European Commission’s *Ethics Guidelines for Trustworthy AI* and the research literature have often suggested that AI should be made ethically acceptable by *increased collaboration* between developers and other stakeholders (Char et al., 2020; Independent High-Level Expert Group on Artificial Intelligence, 2019; Reddy et al., 2020). We agree that this is the preferred way to proceed. As AI technology moves forward, it has become urgent for relevant stakeholders to actively contribute to the translation of broadly acknowledged ethical principles throughout the process of design, implementation, and evaluation. Moreover, for transformative AI technology that reconfigures the conditions of medical practice and leads to abruption with shared normative ideals, such as shared decision-making, stakeholders include everyone affected by healthcare, now or in the future. Thus, in order to ensure AI design that serves patients best, a broad public debate beyond AI designers, bioethicists, and experts might be called upon (Bærøe & Gundersen, 2019; Bærøe et al., 2020).

But how should this collaboration be structured and carried out? A deficit in the literature and policy documents on AI is that the main focus has so far been on the formulation of principles (Mittelstadt, 2019), and there has been less focus on *how* users and designers of AI can apply these principles to shape the use and development of AI (for an exception, see for instance Floridi, 2019). In particular, there have so far been few attempts at providing constructive proposals for *the proper role of professionals, AI designers, and other stakeholders* in applying these principles to the development and use of AI. In order to mend these deficits, the aim of this paper is to provide a systematic discussion of how medical doctors, AI designers, and other stakeholders might help realize ethically acceptable AI in medicine, based on four different models of integrating their input. We refer to these models as the ordinary evidence model, the ethical design model, the collaborative model, and the public deliberation model. Using the obligations of medical doctors derived from the shared decision-making ideal as a normative standard, we provide the basis for a more concrete discussion of why some approaches to ethical AI are insufficient.

## Role Obligations Derived from the Shared Decision-Making Ideal

Rosalind McDougall has recently called for AI technologies in medicine that allow for and facilitate shared decision-making, and concludes that we “need greater dialogue between bioethicists and AI designers and experts to ensure that these technologies are designed in an ethical way in order to ultimately serve patients best” (McDougall, 2019, p. 159). Shared decision-making has been endorsed as the best way to promote trust and well-functioning relationships between doctors and patients since the middle of last century (Kaba & Sooriakumaran, 2007). While including AI within this relationship can improve the clinical outcome, patients can be deprived of their value judgments and treatment options if values become fixed within the AI design (McDougall, 2019). Moreover, introducing AI in the doctor-patient relation might have a negative impact on the trust dimension of the relation (Kerasidou, 2020). Thus, to avoid falling back into paternalistic care where patients are forced to trust blindly either the doctor, the AI system or both, deployment of AI should be aligned with protecting the ideal of shared decision-making.

Shared decision-making can be understood and conceptualized in several ways, but some features are essential. Normative models for the physician–patient relationship highlight the responsibility of the professional to include patients in decision-making. This responsibility encompasses several conditions that must be satisfied for the relationship to be truly inclusive. The doctors must provide adequate information about risks and benefits of treatment options and make sure the patient understands. For example, the information must be based on explainable knowledge, presented in a way that promotes patient understanding. Also, the doctor must ensure that the patients’ values and preferences are explored and taken into account when choosing treatment or preventive care (see, e.g., Emanuel & Emanuel, 1992; Veatch, 1972). For example, people might differ when trading off prospects of some months prolonged life versus burdensome side-effects from medication.

From these general points, we derive four essential role obligations that professionals must accommodate to promote real interaction, communication, and shared discussion (patients must contribute to this process too (see, e.g., Eide & Bærøe, 2021), but to discuss these role obligations falls beyond the scope of this paper):

Doctors must (a) understand the connection between patients’ conditions and the need for potential interventions (this involves both technical and normative considerations) both in general and as translated into the particular contexts of individual patients to identify options, and (b) trust the source of evidence upon which the decisions are to be based (including the reasoning processes involved) to make sure the information is relevant and adequate. Moreover, for doctors to enable patients to participate in the process and share their relevant values and preferences, they must (c) understand all relevant information about benefits and harms and trade-offs between them, and (d) convey it to patients in a clear and accessible manner to ensure that the patients have understood the information and invite them to share their thoughts and deliberate together on the matter. The list is not necessarily exhaustive, but all conditions must, as a minimum, be in place for patients to be justified in trusting that the doctor is aiming for the best outcomes and involving them in doing so. Moreover, as professionals enjoying the discretionary power of being responsible for health care in society, doctors are responsible for ensuring that conditions (a) through (d) are satisfied. If AI systems are to mediate in this relationship between doctors and patients in an ethically acceptable way, they will have to be developed in ways that support shared decision-making. We will now discuss how four different models for medical AI fare in this regard (Table 1).

**Table 1:** The main components of the ideal of shared decision-making

Short description of the components of shared decision-making	Expanded descriptions of the components of what is required of doctors in shared decision-making. These can be perceived as minimum standards	How AI can undermine the conditions for shared decision-making
(a) Understanding the patient’s condition	Doctors must understand the connection between patients’ conditions and the need for potential interventions on a general, technical, and normative level and as translated into the particular contexts of individual patients.	If the clinical outcome of AI is beyond what doctors are able to understand themselves, their clinical competence is undermined, and by that a crucial presupposition for why the patients have reason to trust them in the first place (Kerasidou, 2020).
(b) Trust in evidence	Doctors must base their decision on sources of evidence they trust to make sure the information is relevant and adequate.	If doctors suggest treatments on the basis of AI sources to information they cannot fully account for, they force patients to place blind trust in their recommendations. This is just another version of paternalism.
(c) Due assessment of benefits and risks	Doctors must understand all relevant information of benefits and risk and trade-offs between them.	If doctors cannot fully understand how, and why, AI has reached an outcome, say, a classification of an x-ray, uncertainty regarding assessments of risk, benefits and trade-offs will follow. This, in turn, undermines patients’ reasons to have confidence in their judgments as their role as the expert in the relation.
(d) Accommodating patient’s understanding, communication, and deliberation	Doctors must convey assessment of risks and benefits to patients in a clear and accessible manner, ensure they have understood the information, and invite them to share their thoughts and deliberate together on the matter.	If AI systems makes it hard for doctors to understand how, and why, they reach their outcome, they cannot facilitate patients understanding either. Rather, they will have to paternalistically require that the patient should accept that the AI ’ knows best’.

## Four Models for Medical AI

In this section, we articulate and examine what we take to be four central alternative models of how AI can be designed and applied in patient care, which we call *the ordinary evidence model*, *the ethical design model*, *the collaborative model*, and *the public deliberation model*. While all models carry significant normative insights, we argue that the collaborative model is the most promising for covering most AI technology, while the public deliberation model is called for when the technology is recognized as *fundamentally transforming the conditions* for ethical shared decision-making. Before presenting each of the four models for ethically acceptable medical AI, let us briefly account for the role we attribute to them. First, these models do not primarily purport to represent the ways in which central actors in health care, industry, or academia matter-of-factly see the future ethics of AI in medicine. Rather, the purpose of articulating these four models is mainly to identify central approaches and be able to assess approaches and standards for ethically acceptable AI more clearly. That said, we do think that the four models capture central approaches to ethical AI, and similar ideas can be found tacitly in the literature or, at least, be inferred from what central authors have said about this issue.

As we present them here, the models differ along three main dimensions: (1) the extent to which AI is viewed as a fundamentally transformative technology that calls for new principles, practice, regulation, or governance, (2) the required level of ethical attention among AI designers and users, and (3) the proper division of labor and interaction between AI designers and the medical doctors who use AI in medicine.

## The Ordinary Evidence Model

The ordinary evidence model, as we construe it here, involves two central claims, namely (1) that the output generated by AI amounts to ordinary medical evidence, and (2) that the ethically acceptable use of AI requires that medical doctors (and other health professionals) apply it in a responsible manner using their judgment, medical expertise, and commitment to central principles of medical ethics.

To take the former claim first, “medical evidence” is here understood in a rough-and-ready sense of factual claims based on observations, measurements, research literature, and systematic reviews that can be used to justify significant decisions concerning diagnosis, prognosis, treatment, and prevention of disease. Evidence must satisfy certain standards such as accuracy, reliability, and consistency (which tend to vary depending on the source of the evidence) that provide medical doctors with reasons for making a decision. According to the ordinary evidence model, the use of output from machine learning such as deep neural networks can be integrated into established ways of providing health care services. Medical doctors can apply the output given by the algorithm (e.g., about the probability of cancer in a patient’s sample) the same way they treat other kinds of observations, measurements, and research results as evidence for medical decisions. From this point of view, there is nothing distinct or new about the use of AI in medicine, and its successful implementation is primarily conditioned on its efficiency, reliability, accuracy, and proven effect in clinical trials. The regulatory approval process can follow established procedures. Those AI methods that are applied by doctors in clinical practice must have been proven effective and accurate in clinical trials, approved by regulatory agencies, and introduced to medical practice via some form of training in the use of the method.

Now, turning to the second claim of the ordinary evidence model that we mentioned above, the ethically acceptable use of AI in medicine requires that medical doctors (and other health professionals) apply AI in accordance with *established standards* of medical expertise, ethical guidelines, and laws and regulations. The ordinary evidence model thus fits well with widely held notions of the professional responsibility of medical doctors. A central view of professional decision-making is that medical doctors apply their expertise based on education, training, and clinical experience and act in accordance with their expertise and ethical principles to promote the health of the patients (see for instance, Patel et al., 1999). Given that an algorithm has proven to be accurate and effective, the responsible use of AI in medicine is ensured by the medical doctors’ expertise, judgments, and actions. In other words, algorithms are only assessed according to their epistemic accuracy and instrumental efficiency and not their standards of medical ethics, according to this model.

The ordinary evidence model implies a clear division of labor between the designers of algorithms and medical doctors who are to apply the algorithms in clinical practice. After regulatory approval and successful clinical trials, the algorithms can be sold to health care providers around the world. This means that the design process of, say, an algorithm that detects eye disease or depression is not informed by the medical doctors who use the algorithm. The medical doctors need not have any proper knowledge about the AI methods they apply, the way in which they are designed, and the choices made during that process, and the expertise of the designers and the context of the design. The design process might be culturally *remote* from the medical contexts in which it is applied. The designers need not have any broad medical expertise or familiarity with the doctors’ code of conduct, national regulations, or patients. That said, the ordinary evidence model does not *preclude* the participation of medical doctors in algorithmic design. The point is rather that the contribution of broad medical expertise is not required for proper design.

There are several objections that can be raised to the ordinary evidence model. A first objection is that medical doctors cannot alone ensure the ethically acceptable use of medical AI. An obvious reason for this is that there might be unethical conduct in the process of research and development over which the medical doctors have no direct control; there may also be no due oversight over whether the sources of evidence should be trusted (cf. condition b) of the ideal of shared decision-making above). For instance, if the design of algorithms has violated the privacy of those patients whose data are used for training of algorithms, it will not suffice for doctors to apply them in a clinically adequate manner. However, the ordinary evidence model faces more fundamental objections. Indeed, most of the *distinct ethical concerns* over medical AI in the literature over such things as risk of error, discrimination due to algorithmic bias, problems with accountability, and lack of transparency can be formulated as objections to the ordinary evidence model. In short, the ordinary evidence model does not provide a reasonable normative response to the ethical challenges that applied AI raises for any professional practice. To see this, let us consider one of these concerns: an accountability problem that the use of AI can cause in medicine.

The ordinary evidence model states that the ethical standards of medical AI are ensured by the responsible conduct of doctors according to the standards of medical expertise and ethical principles. This presupposes that medical doctors can be properly held to account when using AI in their clinical practice. There are two main ways in which medical AI poses a problem for accountability. One source of the accountability problem is structural and concerns the difficulties in ascertaining to whom praise and blame can be attributed. In the case of errors such as misdiagnosis based on false evidence generated by AI, it is not clear who should be blamed. Is it the medical doctor who made the diagnosis using AI, the institution in which the doctor works that decided to apply that method, the computer scientists who designed the algorithm, or the algorithm itself?

Another problem for doctors’ accountability stems from the opacity of AI. If medical doctors are unable to fully understand the processes behind the output of machine learning algorithms upon which they base their decisions in clinical practice—for instance, whether it involves relevant uncertainties, biases, and privacy threats—it will be difficult for them to give proper account to patients as essential to shared decision-making. Opacity challenges the professional role obligation of the ideal of shared decision-making involved in translating general medical knowledge into particular cases, i.e., as in (a) above, on inaccessible reasoning, since the relevant factors in the particular situation (which can become implicitly or explicitly known to the doctor through experiences) remain inaccessible if processed by machine learning. Moreover, opacity challenges the conditions of ensuring sources of evidence that can be trusted, making due assessment of risks and benefits, and even engaging in clear communication with patients—i.e., conditions (b) through (d)—too. In sum, the reliance on AI in medicine challenges and even disrupts the professional accountability upon which the ordinary evidence model rests.

## The Ethical Design Model

Our discussion of the ordinary evidence model suggests that the use of AI in medicine raises ethical problems that cannot be solved by medical doctors’ responsible use alone. A reasonable response to the fact that AI has ethically problematic consequences, then, is to improve the very process of AI design. According to the ethical design model, the ethical use of AI in medicine requires that algorithms be designed in an ethical way by encoding ethical values directly into them. Indeed, much work in AI research and machine ethics currently revolves around this approach (for an overview, see Misselhorn, 2018). In a recent book, *The Ethical Algorithm. The Science of Socially Aware Algorithm Design* (2020), computer scientists Michael Kearns and Aaron Roth argue that the most promising approach to avoiding harm to people as a result of the use of machine learning algorithms is found in “the emerging science of designing social constraints directly into the algorithms, and the consequences and trade-offs that emerge” (p. 16). In their view, the “science of ethical design” avoids the problems of traditional approaches of new laws and regulations (such as the General Data Protection Regulation of the EU) “to enforce still-vague social values such as ‘accountability’ and ‘interpretability’ on algorithmic behavior” (p. 15). Their approach is rather understood as “the new science underlying algorithms that internalize precise definitions of things such as fairness and privacy—specified by humans—and make sure they are obeyed. Instead of people regulating and monitoring algorithms from the outside, the idea is to fix them from the inside” (pp. 16–17). To apply this principle to medicine, AI designers could, then, implement widely shared ethical principles in the machine learning algorithms, which would then ensure that their output is ethically acceptable in medical contexts.

This model conveys some reasonable claims. Above all, this approach takes the distinct ethical challenges of applying machine learning algorithms seriously. The ethical design model is a reasonable response to the lack of ethical attention in AI design. Moreover, some of the ethical problems that are raised by AI in medicine require that AI designers play an active role. A case in point is respect for the privacy of patients who have generated the data or the securing of access to the data; this must be handled in the design process. If the design of algorithms disregards the right to privacy of patients, using algorithms so developed cannot be ethically defensible. Moreover, algorithmic bias is a recurring problem. Algorithmic bias can be caused by skewed data that result from the fact that some groups are underrepresented in the available data or that the designers are biased when selecting data (for a detailed taxonomy of algorithmic bias, see Danks & London, 2017, see also, Suresh & Guttag (2021). Algorithmic bias can lead to discrimination against certain social groups due to their gender, ethnicity, and sexual preference. While it is difficult to remove vectors that contain information that will yield such biases, AI designers have developed concrete techniques for approximating fairness in design (for examples, see Kearns & Roth, 2020, chapter 2).

Even though the ethical design model might generate more ethically attentive design, it faces several challenges. It entails a problematic division of labor between designers and medical doctors, which generates a set of problems pertaining to a lack of fit between design and medical practice. Let us point to some problems that the strict division of labor between AI designers and medical doctors can lead to.

First, according to the strong interpretation of the ethical design model, ethical design is sufficient for the ethically acceptable use of AI. It implies that AI can be ethically acceptable independently of how it is used (cf. the concept of “the ethical algorithm”). This means that in so far as values such as privacy and fairness are encoded directly into the algorithm, ethically acceptable medical practice is ensured. Indeed, both Kearns and Roth and the field of machine ethics focus on algorithms as ethical subjects with the capability of making ethical choices to avoid harm. While this view does not necessarily mean that the moral judgment of medical doctors will become superfluous, it goes as far as to imply a technocratic view in which ethical choices are made by experts (see Jasanoff, 2016 for an interesting discussion of technocracy in the context of technological innovation). Moreover, the choices made in the design process of which values to encode into the algorithms might make it difficult for medical doctors to bypass or overturn these choices. For instance, if the algorithms are designed to detect disease in its early stages or in less clear cases, this might lead to an overly high instance of false positives that cannot be counteracted and corrected by medical doctors.

In our view, while some ethical problems can be addressed in the design process, they cannot alone make AI ethically acceptable in medicine. By applying algorithms that have undergone the “science of algorithmic design,” practitioners might be led to think that further ethical reasoning and deliberation is superfluous. By implication, the goal of designing ethical algorithms removes a central part of ethically relevant reasoning among doctors—for instance, about such things as the distribution of false positive and false negatives as part of the harm assessments, whether the observed symptoms warrant further examination due to uncertainty, whether the patient should receive this or that treatment, or whether other factors in the patients’ lives besides the analyzed data are relevant to further treatment. It could create the misconception that once the ethical design of AI is in place, then the implementation of that technology can proceed seamlessly in a responsible manner. If so, ethical design might entail the outsourcing of ethical deliberations by medical practice to AI designers; in such a case, the ethical structural condition (c) of due assessment of risk and benefits and consequently (d) of accommodating for patient’s understanding, communication, and deliberation may not be obtained. When this happens, it undermines the conditions for realizing the shared decision-making ideal.

Second, ethical design involves the formalization of the ethical values encoded into the algorithms. Therefore, to the extent that algorithms can be made ethically acceptable, values such as privacy, fairness, veracity, and accuracy must be formalized. Such formalization raises several difficult problems, some of which Kearns and Roth are aware of, but they do not include a promising approach for solving them. In our view, the ethical design model downplays the need for specification and translation of ethical values in concrete cases. While most people are committed to fairness in public health care, it is open to debate exactly how fairness should be understood (this goes for both substantive versions of distributive fairness and procedural fairness) and how it applies to concrete cases where medical doctors make crucial decisions concerning their patients. Values and principles such as veracity, accuracy, transparency, and accountability are partly constituted by humans interpreting them and balancing them in concrete cases. Given the fact that values must be interpreted to make sense in a concrete case, it seems misguided to claim that ethical designs made at a distance from the context of application are comprehensively justified. The ethical structural condition (a), which underscores the doctor’s ability to translate general knowledge of normative concerns into the specific circumstances of individual patients, is not satisfied. Thus, shared decision-making is undermined by the formalization of ethical values encoded into algorithms.

We draw two important lessons from our discussion of the ordinary evidence model and the ethical design model. First, the discussion so far points to the need for ethical deliberation in design *and* use. While it is reasonable for designers to take ethical considerations carefully into account, this should not exempt doctors from critically assessing the design process and the algorithms’ appropriateness in use. Second, in both models, the division of labor between algorithmic designers and medical doctors who apply the algorithms becomes too strict. Most important, in addition to being procedurally legitimate in terms of respecting privacy and enabling professionals to give account, medical AI must be substantially informed by the code of conduct of health professionals who have direct experience with ethical problems. The collaborative model provides us with reasonable ways to deal with these two problems.

## The Collaborative Model

The collaborative model states that collaboration and mutual engagement between medical doctors and AI designers are required in order to align algorithms with medical expertise, bioethics, and medical ethics. Indeed, this model aims to bridge the gaps between AI designers and medical doctors in terms of their expertise and their commitment to ethical principles. The collaboration model comprises two main claims. First, it states that there must be collaboration between designers and doctors, as well as expertise in ethics, in both the design and use of medical AI. Second, AI designers, bioethicists, and medical doctors must have the capacity to communicate meaningfully about the way algorithms work, their limitations, and the algorithmic risks that arise in clinical decision-making. In order to clarify the collaborative model, we shall here explicate the nature and scope of collaboration. Moreover, we shall argue that fruitful collaboration is conditioned on a set of competencies.

Let us here suggest three ways in which such collaborations could be realized. First, medical doctors can be an *active part of the design of medical AI*. In fact, there seems to be a de facto commitment to such collaboration in ongoing research and development in medical AI. Both in academic research institutions and industry, the design process is often informed by medical expertise. Doctors and designers can collaborate in the initial stage of research and development by identifying what medical specialties or tasks might benefit from AI assistance. Existing studies examine the accuracy and efficiency of deep learning by testing how well algorithms perform a specific task, such as identifying cancer in pictures, in comparison to the performance of clinicians, without examining whether the clinical use of the algorithms leads to better health care services and improved health for patients (Topol, 2019a). Since there are few studies examining the effect on clinical practice, AI designers and doctors could set up proper clinical trials and studies.

Based on their medical expertise and experience of communicating with patients about patients’ needs and values, medical doctors can, together with bioethicists with training in ethical theory and analytical discrimination of normative concerns, inform algorithmic designers about what parts of decision-making require patient involvement and individual trade-offs. Medical doctors could communicate to designers what levels of accuracy are needed for specific tasks and the trade-offs between principles and standards in real-time decision-making, for instance between accuracy and urgency in emergency situations. Moreover, medical doctors could play a vital role in properly calibrating the algorithms’ rates of false positives and false negatives. Given the fact that some algorithms have proven to have unacceptably high rates of false positives, doctors could provide useful input to the design process about the importance of reducing the algorithm’s “eagerness” to detect signs of disease in some cases.

Second, AI designers can engage with medical doctors to better *understand and interpret AI output in a reasonable manner in clinical practice*. As we have shown above, given the lack of understanding of how deep learning algorithms work, these limitations should be taken into consideration when applying algorithms in decision-making. When deep learning algorithms provide an analysis of data, for instance by classifying a patient’s data as an indication of pneumonia, medical doctors must be able to properly interpret the algorithm’s output—for instance, what it means that the algorithm states that there is a 70% probability of the patient having pneumonia, the algorithm’s distribution of false positives and false negatives, and the reliability of the algorithms in the face of outliers and novel phenomena.

Regarding the second claim of the collaboration model, fruitful collaboration between designers and doctors is conditioned on their capacity to communicate across their domain of expertise. On the one hand, algorithmic designers must be aware of the ethical aspects of the algorithms they develop and be well informed about medical expertise and the ethical guidelines that regulate medical practice. On the other hand, medical doctors who use AI in medicine must be well informed about how the algorithms work and the uncertainties and limitations of AI output. They should also be able to explain to patients how an AI analysis has been performed and how it has informed their decision about a diagnosis or treatment plan. In sum, medical doctors must play a role in the design process in order to enhance both the *medical literacy* of AI developers and the *AI literacy* of medical doctors (for a relevant discussion of the notion of expert literacy, see Eriksen, 2020).

Finally, a third way in which collaboration between designers and other experts can be realized is through *evaluating the impact of AI on clinical practice*. If AI is going to inform crucial parts of medical decision-making in the future, it is vital that medical doctors who apply AI as a part of their clinical practice evaluate the impact of AI on decision-making and share their assessment with colleagues and AI designers. We shall not go into detail here about how such evaluation should be performed and governed. Our main point is merely that there must be established avenues for criticism, objections, and suggestions from clinicians and bioethicists to designers.

Now, how is the collaborative model able to avoid some of the ethical problems that we have discussed so far? Collaboration and mutual capacity of communication between AI designers, bioethicists, and medical doctors avoid some of the problems that stem from viewing AI outputs as standard medical evidence. If medical doctors understand the way the output is generated and its reliability, both the input from AI and doctors’ understanding and assessments will become more transparent, thus alleviating some of the accountability problem and satisfying conditions (a) through (c) for shared decision-making. This applies particularly to the ability of medical doctors to give proper accounts to patients in the case of error by explaining why AI is being used, how it works, its known limitations, and its possible causes of errors. A crucial issue here, concerns how medical doctors can gain the required competence in AI. While we cannot go in detail about this issue here, the use of AI in medical decision-making should be taken into consideration by higher education institutions when designing the curricula for medical doctors and other health care professionals. Moreover, since this technology is new and evolving, it seems reasonable for universities, public health institutions, industry, and medical associations to collaborate on developing courses for medical doctors (for a very interesting discussion of this issue, see Quinn et al., 2021).

The medical literacy of AI developers and the AI literacy of medical doctors can enable doctors to promote shared decision-making’s emphasis on sources of evidence deemed to be trusted, due harm assessment, and proper communication. While we find the collaborative model promising, we will now briefly point to the need for a fourth model in light of the high risk of AI in healthcare of reducing inter-human encounters and communication.

## The Public Deliberation Model

The use of AI involves what we call *meta-ethical risks* that arise from a lack of inter-human encounters, experiences with human vulnerability, and deliberation. By “meta-ethical risks,” we refer here to circumstances that may pose a challenge to conditions for practical ethics within a human-intelligence-centered worldview (as we are familiar with today). To the extent that AI technologies lead to a decrease in required communication and exchanges of information between doctors and patients, we face the risk of undermining the ideal of shared decision-making. Moreover, while leaving some of the communicative examination work to algorithms may produce more effective health care, it can also undermine professionals’ engagement with patients’ social, emotional, and existential challenges (Bærøe & Gundersen, 2019). Compassion, empathy, solidarity, and recognition of injustice may arise in such encounters and in turn influence motivation, actions, practical ethics, and political ideology. Such meta-ethical conditions for ethics in practice may be fundamentally changed if the social conditions for interaction and shared decision-making in healthcare are increasingly replaced by AI technology.

In our view, both the threats of undermining the ethical conditions for shared decision-making and meta-ethical conditions driving ethics in medicine in general “as we know it” might be considered unavoidable risks of applying AI in medicine. However, deciding on designing and employing technology with such disruptive, transformative impacts should not be left to AI designers, bioethicists, and medical expertise alone; it calls for broad public debate about whether the costs and risk of AI are outweighed by its potential benefits for patients and society at large. The public deliberation model involves more stakeholders than AI designers, bioethicists, and medical experts. It includes policymakers and the general public, too. When agents in the cooperation model screen for and identify the fundamentally transformative impact of a new AI technology, the public deliberation model is required. It is beyond the scope of this paper to discuss the details of how this deliberation should be organized. We will therefore simply point out that a reasonable, general expectation is that it should be carried out in correspondence with conditions of democratic governance. This view is also compatible with the EU report on how trustworthy AI requires public deliberation (Independent High-Level Expert Group on Artificial Intelligence, 2019), but much more work is required to protect the ethics of—and within—such broad, public, shared decision-making processes (Table 2).

**Table 2:** The four models

	Transformative and disruptive technology	Ethical attention	Division of labor	Benefits	Challenges
Ordinary evidence model	No	Mainly in use, not design	Distinct	Fits well with widely held notions of the professional responsibility	Lacks proper response to challenges pertaining to algorithmic risk, transparency, and accountability
Ethical design model	Yes	Mainly in design, not use	Distinct	Takes the distinct ethical challenges of medical AI seriously	Technocratic view on ethical choices and the problem of formalizing ethics
Collaborative model	Yes	Both in design and use	Integrated	Alleviates some of the accountability problem and promotes shared decision-making	No proper response to severe ethical risks
Public deliberation model	Yes	Both in design and use, and the public sphere	Partly distinct, partly integrated	Can deal with “meta-ethical risks”	The models need more organizational specification

## Conclusion

In this article, we have argued that the ordinary evidence model and the ethical design model downplay the fact that AI involves ethical value judgments in *both* design and application. The clear division of labor between designers and doctors, which both models imply, has problematic consequences in terms of not aligning AI with medical expertise and medical ethics and by not enabling medical doctors to properly understand the way in which the algorithm is designed and its limitations. The collaborative model alleviates these problems by emphasizing the need for including medical doctors and bioethicists in algorithmic design and improving their AI literacy in the context of application. However, this does not mean that the collaborative model solves all the central ethical challenges raised by the use of AI in medicine. AI technology that can increase effectiveness and precision but may disrupt conditions for human-intelligence-centered ethics and undermine ethical ideals, like shared decision-making, calls for broader deliberation over value trade-offs involved in the development and use of AI in health. The public deliberation model captures the broader social processes of including the public beyond AI designers, medical experts, and bioethicists. Further work is clearly required to carve out the distinct roles of AI designers, medical and ethical experts, policymakers, and the general public in developing AI for health.

Our contribution in this paper is both systematic and substantial. In regard to systematicity, we provide an account of the central ways in which medical doctors, bioethicists, and designers can make AI ethically acceptable that we find to be lacking in the current AI ethics literature more generally and in the case of medical AI. By articulating four models, we enable a more systematic discussion of how different kinds of ethical concerns can be approached in medicine by central actors. In regard to our substantial contribution, our discussions of the distinct models purport to provide the future of medical AI *not only with principles, but also a proposal* (Estlund, 2019, p. 10) for *how* central actors can contribute to making medical AI ethically acceptable by interaction, mutual engagement, and their competencies.
